# Atraumatic Bi-femoral Axillary Bypass Graft Non-anastomotic Disruption With Pseudoaneurysm Formation Detected by Doppler Ultrasound

**DOI:** 10.7759/cureus.30871

**Published:** 2022-10-30

**Authors:** Danielle A Nesbit, Carrie Wang, Phillip M Grenz, Kevin R Roth, Jessica K Eygnor

**Affiliations:** 1 Department of Emergency and Hospital Medicine/University of South Florida Morsani College of Medicine, Lehigh Valley Health Network, Bethlehem, USA

**Keywords:** graft complication, atraumatic, non-anastomotic, pseudoaneurysm, vascular graft

## Abstract

Bi-femoral axillary bypass graft placement is a well-known and typically safe procedure. It is generally indicated for patients with advanced peripheral vascular disease, aortoiliac occlusive disease, or infectious artery disease. In rare cases, the graft can be fractured or dislodged after placement, though most often, this occurs almost exclusively at the anastomosis site, secondary to blunt trauma. Using ultrasonic imaging is a reliable method of detecting these fractures. We present a case of a bi-femoral axillary bypass graft fracture in a 68-year-old male with the development of a pseudoaneurysm in the right lateral abdominal wall. The patient reported spontaneous development of a “strange” sensation in his right lower abdomen and a “painful lump” upon waking. Physical examination showed a small right lower quadrant outpouching which was pulsatile on palpation. The initial workup included a bedside ultrasound which showed a fractured graft with the fluid collection and a Doppler signal. Vascular surgery was immediately consulted for evaluation, and the patient was taken to the operating room for emergent surgical repair. CT angiography confirmed a successful operation in which an 8 mm graft was placed to anastomose the original bypass graft fracture site. The patient remained stable postoperatively and was discharged without further complications. This report highlights the importance of using ultrasonography for the immediate identification of potential graft complications to prevent serious complications and expedite definitive management.

## Introduction

Polytetrafluoroethylene (PTFE) grafts have been used by vascular surgeons for decades to improve blood flow in patients that suffer from conditions that occlude or otherwise impair their native circulatory systems [1]. Of particular interest, in this case, is the bi-femoral axillary bypass (BiFAXB) graft which is performed on patients with peripheral vascular disease, aortoiliac occlusive disease, or infectious artery disease [2]. Those afflicted by these diseases tend to be older and may have other comorbidities that can impact their odds of survival and graft patency [1,2]. In the setting of peripheral vascular disease, there is some debate as to the graft’s 5-year patency rate, with rates ranging from 54% to 80.4% [3,4]. Due to its relative risk when compared to other treatment options, BiFAXB graft placement is typically used as a final line of treatment for those with severe vascular disease [2].

While there are other potential complications associated with BiFAXB graft placement, the most common of which are thrombosis and infection, one of the most acutely life-threatening is graft disruption or fracture [5]. In general, a graft disruption will occur at the anastomosis site and can result from a variety of causes, the most common being trauma or infection [5]. Non-anastomotic graft fracture is extremely rare and can typically be directly attributed to a specific etiology [5]. Having a discrete incident that leads to symptom development can help physicians to narrow down their diagnostic differential much more rapidly, thus allowing for a quicker transfer to higher levels of care [6]. Without such an event leading up to presentation, physicians must instead rely on symptoms, lab results, and imaging to elucidate the underlying condition [6]. In the case of vascular graft fracture, this must be done rapidly to increase the patient’s odds of survival [7].

Point-of-care ultrasound (POCUS), including Doppler ultrasound, remains a fast, efficient, and crucial tool in the prompt diagnosis of many time-sensitive clinical conditions. Previous studies have found that POCUS improves both the accuracy and speed of diagnosis in acute respiratory and cardiovascular conditions, as well as improving understanding between patients and providers [8,9]. POCUS, additionally, may play an essential role in early diagnosis of rarer conditions such as graft disruption, as seen here, and potentially other, less common post-surgical complications. Only a few cases of non-anastomotic axillary bypass graft disruption have been previously described in the literature [5,10,11]. The vast majority of fractures are in PTFE grafts secondary to blunt trauma [5,9,10]. We discuss a patient who presented with atraumatic BiFAXB PTFE graft disruption and the impact bedside POCUS had on both the diagnosis of this unique condition and expedition of patient care.

## Case presentation

A 68-year-old male presented to the emergency department complaining of a newly developed “lump” with right lower quadrant abdominal pain. His past medical history included peripheral vascular disease and a BiFAXB using PTFE interposition grafts 6 months prior. His medications at the time included apixaban, metoprolol, ramipril, rosuvastatin, and sildenafil.

**Figure 1:**
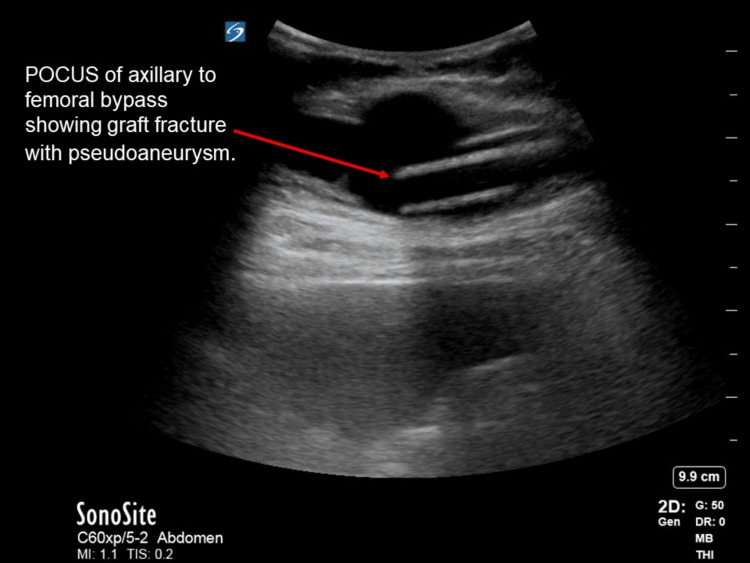
Sagittal and sonographic image of the vessel with the graft fracture.

**Figure 2:**
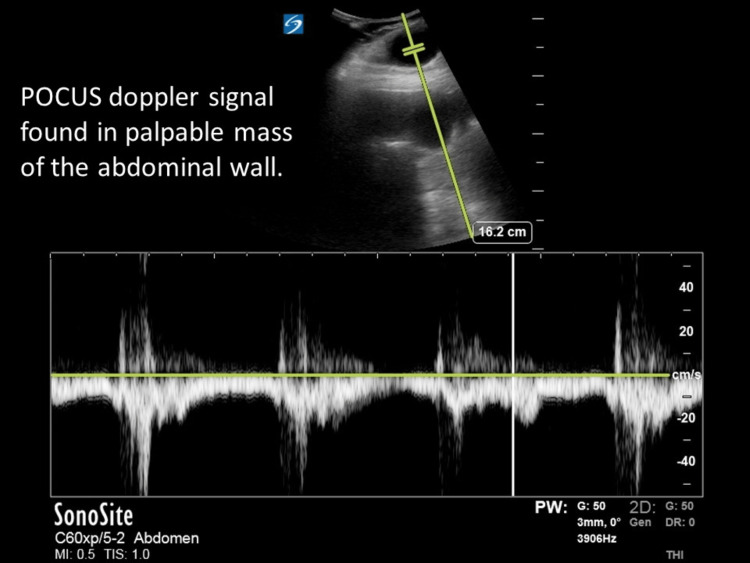
POCUS Doppler showing pulsatile flow in abdominal mass. POCUS: point-of-care ultrasound

The patient denied any trauma or injuries, stating that the previous night he felt a “weird feeling” that was not painful. Upon waking, he shifted onto his right side and felt a “popping” sensation; a painful lump subsequently developed in the lower right abdominal quadrant after which he decided to come to the ED. The initial physical examination revealed a bulge in the right lateral abdomen and a hernia was initially considered as a key differential. However, on palpation, the bulge was found to be pulsatile. The patient had faint dorsalis pedis and tibialis posterior pulses bilaterally with a Doppler signal present, and extremities were warm with no signs of ischemia. Bedside abdominal POCUS was performed which showed evidence of fluid collection with a pulsatile Doppler signal as well as an area of echogenicity which was assumed to be the graft terminating within the fluid collection area (Figures 1-2). On CT imaging, the fluid collection area measured 4.4 x 2.8 x 4.7 cm.

**Figure 3:**
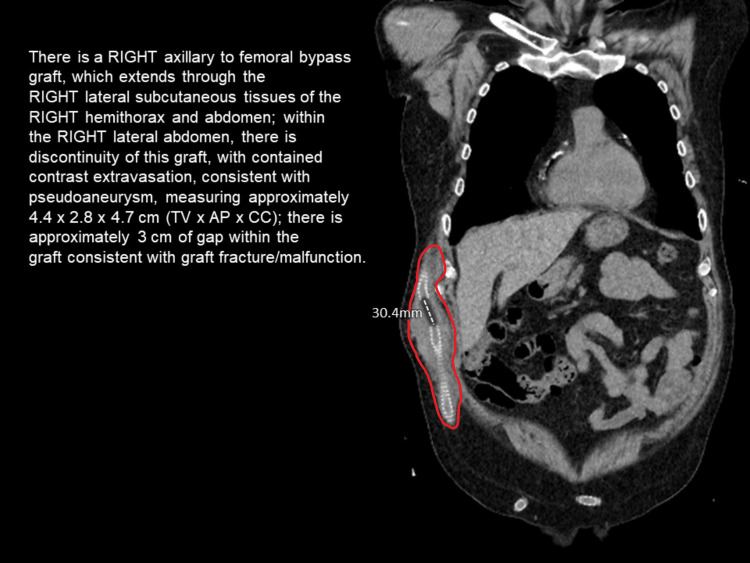
CT angiogram showing graft fracture with pseudoaneurysm and active extravasation. The red outline indicates the boundaries of the pseudoaneurysm.

POCUS findings provided quick evidence of graft fracture with subsequent pseudoaneurysm. Given this, vascular surgery was emergently consulted for evaluation and definitive management. Urgent CT angiogram preoperatively confirmed the diagnosis of a fractured graft in the mid-lateral right abdomen with superficial hematoma development and active extravasation (Figure 3).

Lab results showed slightly decreased hemoglobin (12.4 g/dL, ref range: 12.5-17.0), hematocrit (35.9%, ref range: 37.0-48.0), red blood cell count (3.53 mill/mm, ref range: 4.0-5.4), and mean platelet volume (7.2 fL, ref range: 7.5-11.3). An EKG performed in the ED showed sinus bradycardia with occasional premature ventricular complexes. A pressure dressing was applied to the hematoma, and the patient was taken to the OR for exploration and repair prior to CT results. Per our discussion with vascular surgery and radiology, this was an extremely rare complication of vascular graft surgery, especially given the non-anastomotic location of the graft fracture. With the aid of the bedside POCUS examination demonstrating our findings, we were able to promptly diagnose this complication and quick bedside imaging was useful in reducing delay in specialist evaluation and definitive care.

## Discussion

BiFAXB grafts allow for adequate blood flow in patients with obstructive blockage of the abdominal aorta or iliac arteries with subsequent lower limb ischemia. A flexible plastic tube is placed to divert blood from the axillary artery directly to the femoral arteries, bypassing the areas of blockage. This procedure is indicated for patients with peripheral vascular disease, aortoiliac occlusive disease, or infectious artery disease [11,12]. In a comparative study, Passman et al. examined the morbidity, mortality, and patency rates for aortofemoral bypass grafting and BiFAXB grafting. BiFAXB was found to have a significantly lower postoperative complication rate than aortofemoral bypass grafting, though the two procedures were of similar risk and effectiveness in all other categories that were studied [3]. As a result, BiFAXB was selected to treat this patient’s peripheral vascular disease, as it carries the lowest risk of complication, compared to similar treatment modalities.

Despite this, typical post-surgical complications include local wound infection, seroma or hematoma formation, surgical site pain, graft thrombosis, and arterial steal syndrome [5]. Non-anastomotic graft fracture, however, is an extremely rare complication with few previously reported cases [13]. The majority of graft fractures are in PTFE grafts at an anastomosis site secondary to acute blunt trauma [5]. A single 2008 case report by Grochow and Raffetto reported a graft fracture due to chronic, repetitive, low-grade trauma [14]. This makes our patient’s atraumatic, non-anastomotic graft fracture unique, and the exact cause of the complication remains unclear.

In the absence of a causal event, a wider differential diagnosis had to be considered after the patient presented to the ED. While this patient’s hematologic lab results showed mildly decreased values, they were all very close to normal ranges and there was no sign of infection. As such, the cause of the newly formed pulsatile hematoma had to be investigated using imaging to discover its origin. Using POCUS Doppler imaging, we were able to discover the origin of the hematoma more rapidly than a CT angiogram study could be performed. Consequently, the patient was able to receive emergent care in less time than if POCUS had not been used. The patient went on to have a complication-free recovery and is doing well today.

## Conclusions

In this case, a patient presented with a rare surgical complication resulting from a unique cause and atypical symptom presentation. The patient’s history taken upon admission to the ED did not indicate an immediate concern for graft disruption, as he reported no recent traumatic events or strenuous activity. Bedside POCUS allowed for quick identification and diagnosis of this emergent post-surgical complication. POCUS provided immediate evidence of this extremely rare condition which allowed for vascular surgery transfer for definitive operative management without waiting for a CT angiogram. Utilization of fast, efficient, and cost-effective POCUS allows for rapid detection of emergent medical conditions where even the slightest delay in these clinically tenuous scenarios can impact patient morbidity and mortality. In this case, it provided an almost immediate diagnosis of vascular graft fracture with extravasation and allowed for more rapid transfer to the operating room for repair of the graft.
